# Undocumented children’s health – key challenges and local responses

**DOI:** 10.1186/1824-7288-40-S1-A47

**Published:** 2014-08-11

**Authors:** Michele LeVoy, Lilana Keith

**Affiliations:** 1 PICUM, Platform for International Cooperation on Undocumented Migrants, Brussels, 1000, Belgium

## Introduction

Every European Union member state has legal obligations to protect the rights of the child. Nevertheless, there are children in Europe that face significant barriers to accessing even essential health services, due to their or their parent’s migration or residence status. Undocumented children usually receive health care under the same conditions as adult undocumented migrants, with no extra protection, and are often unable to access the majority of the health care services they need.

## Access to health care in law and practice

National laws regarding access to health care for undocumented children– what care children may access and under what conditions and costs - vary enormously across Europe. In many countries, undocumented children are only able to access emergency health care services, and in a few, even this many be subject to charging. In a number of countries, certain types of care that are considered ‘urgent’, ‘essential’ or ‘medically necessary’ are provided. In a number of others, all children are afforded equal or near equal access to health care services [1-3].

Numerous practical barriers can affect children’s ability to access to care that they are entitled to. These include discretion on local level, varied interpretations of what care undocumented children are entitled to, lack of awareness of children’s entitlements, and fear of detection/ denunciation [3-5].

Children’s health may also suffer generally due to a lack of continuous care, lack of access to specialist and mental health care, and due to conditions related to living in an irregular migration situation, such as poor housing conditions and stress [6,7].

## Local responses

Non-governmental organisations, individual health professionals and in some cases, local and regional hospitals and health authorities are striving to fill the gaps left in state service provision and meet urgent health needs in their communities despite limited resources, while also advocating for policy change [8-10].

## Conclusion

It is the duty of governments to ensure non-discriminatory access to health care services. There is a growing body of evidence that indicates that universal health coverage is necessary and beneficial from the perspectives of individual health, development and social inclusion, public health (including functioning and financing of public health systems), human rights, medical ethics and humanitarian principles. Alongside the examples of inclusive practice from national, regional and local levels, this evidence base requires expeditious reform of discriminatory migration and health policies.
